# Does Chronic Kidney Disease Really Affect the Complications and Prognosis After Liver Resection for Hepatocellular Carcinoma? A Meta-Analysis

**DOI:** 10.3389/fsurg.2022.870946

**Published:** 2022-04-06

**Authors:** Xiao-Yu Liu, Zhi-Qiang Zhao, Yu-Xi Cheng, Wei Tao, Chao Yuan, Bin Zhang, Chun-Yi Wang

**Affiliations:** ^1^Department of Gastrointestinal Surgery, The First Affiliated Hospital of Chongqing Medical University, Chongqing, China; ^2^Department of General Surgery, Qijiang Hospital of the First Affiliated Hospital of Chongqing Medical University, Chongqing, China

**Keywords:** chronic kidney disease, hepatocellular carcinoma, overall survival, complications, meta-analysis

## Abstract

**Purpose:**

The purpose of this meta-analysis was to analyze whether chronic kidney disease (CKD) affected the complications and prognosis after liver resection for hepatocellular carcinoma.

**Methods:**

The PubMed, Embase, and Cochrane Library databases were searched from inception to 22 February 2022 to find eligible studies. Complications, overall survival (OS), and disease-free survival (DFS) were collected, and this meta-analysis was performed with RevMan 5.3.

**Results:**

A total of nine studies including 6,541 patients were included in this meta-analysis. After pooling all baseline information, the CKD group had a higher rate of Child-Pugh grade B than the Non-CKD group (OR = 1.58, 95% CI = 1.3 to 1.93, *P* < 0.00001). As for surgery-related information, the CKD group had larger blood loss (MD = −404.79, 95% CI = −509.70 to −299.88, *P* < 0.00001), and higher rate of blood transfusion (OR = 2.47, 95% CI = 1.85 to 3.3, *P* < 0.00001). In terms of complications, the CKD group had a higher rate of overall complications (OR = 2.1, 95% CI = 1.57 to 2.81, *P* < 0.00001) and a higher rate of ≥ grade III complications (OR = 2.04, 95% CI = 1.57 to 2.81, *P* = 0.0002). The CKD group had poor OS compared with the non-CKD group (HR = 1.28, 95% CI = 1.1 to 1.49, *P* = 0.001). However, in terms of DFS, no significant difference was found (HR = 1.11, 95% CI = 0.96 to 1.28, *P* = 0.16).

**Conclusion:**

Preexisting CKD was associated with higher ratio of complications and poor OS.

## Introduction

Hepatocellular carcinoma (HCC) is the fifth most common malignant tumor and the fourth most common cause of cancer-related deaths in the world (1, 2). It is estimated that 840,000 newly diagnosed HCC cases are reported each year worldwide (3). Liver resection is commonly radical treatment (4, 5).

Chronic kidney disease (CKD) is a serious public health problem in the 21st century (6) and leads to premature death, reduced quality of life, and heavy economic burden on patients (7). The morbidity and mortality of CKD are increasing rapidly worldwide (8), and compared with the non-CKD population, the morbidity and mortality usually come from cardiovascular disease (9, 10).

Previous studies have reported the relationship between CKD and cancer surgeries, and CKD increased complications and mortality (11, 12). Multiple systems are impaired with progression of CKD (11), which might account for higher complications and poorer overall survival.

As for HCC, CKD might have a potential effect on outcomes. However, it remains controversial whether preexisting CKD has an impact on complications and prognosis after liver resection for hepatocellular carcinoma. Some studies reported that CKD had no effect on complications or prognosis; (13, 14) however, other studies reported that CKD was associated with increased complications and poor prognosis (15, 16). Therefore, the purpose of this meta-analysis was to analyze whether CKD affected the complications and prognosis after liver resection for hepatocellular carcinoma.

## Methods

### Literature Search Strategy

The PubMed, Embase, and Cochrane Library databases were systematically searched by two authors, and searching date was 22 February 2022. The search strategy mainly focused on the following two items, HCC and CKD. For HCC, the search strategy was as follows: (liver cancer) OR (hepatocellular carcinoma) OR (hepatocarcinoma) OR (HCC); for CKD, the search strategy was as follows: (chronic kidney disease) OR (hemodialysis) OR (estimated glomerular filtration rate) OR (dialysis) OR (cystatin c). Then, we used “and” to combine two key words. The scope of the search strategy was limited to the Title and Abstract, and the language was limited to articles published in English.

### Inclusion and Exclusion Criteria

The inclusion criteria of this meta-study were as follows: (1) Patients diagnosed with HCC and received primary liver resection; (2) The preexisting disease included CKD group and Non-CKD group; and (3) Postoperative short-term complications or long-term prognosis were reported. The exclusion criteria were as follows: (1) data that could not be extracted in the meta-analysis; and (2) research type was case report, letter to editor, comments, reviews, or and conference. Two reviewers conducted the inclusion and exclusion separately, and a disagreement was resolved by another reviewer.

### Data Extraction

The extracted data were as follows: (1) first author, year of publication, country, study design, study date, definition of CKD; (2) baseline information including sex, age, tumor size, vascular invasion, intrahepatic metastases, Child-Pugh grade, and liver cirrhosis; (3) surgery-related information including blood loss, blood transfusion, and postoperative hospital stay; (4) postoperative information including overall complications, surgical site infection, bile leakage, liver failure, pleural effusion, ascites, and postoperative bleeding; and (5) overall survival (OS) and disease-free survival (DFS).

### Quality Assessment

To assess the methodological quality of included retrospective studies, we adopted the Newcastle-Ottawa Scale (NOS) (17)If the NOS score was equal to nine points, we considered the methodological quality of a study to be high; if the NOS score was less than seven points, we considered the methodological quality of a study to be low; if the NOS score was between seven and eight, we considered the methodological quality of a research study was moderate.

### Statistical Analysis

The meta-analysis was performed using RevMan 5.3 (The Cochrane Collaboration, London, United Kingdom). In this meta-analysis, pooled hazard ratios (HRs) and 95% confidence intervals (CIs) were calculated for the OS and DFS of patients with hepatocellular carcinoma, and HRs were extracted from multivariate analyses and/or univariate analyses, or estimated from Kaplane-Meier survival curves (18, 19). Continuous variables were presented as the mean and standard deviation (SD), and categorical variables were presented as proportions. For dichotomous and continuous variables, odds ratios (ORs) and mean differences (MDs), and 95% CIs were calculated. The I^2^ value and the results of the chi-squared test were used to assess statistical heterogeneity (20, 21). High heterogeneity was considered when I^2^ > 50%; in such a case, a random effects model was used, and *p* < 0.1 was considered statistically significant. A fixed effects model was used when I^2^ ≤ 50%, and *p* < 0.05 was considered statistically significant.

## Results

### Study Selection

**Figure 1:**
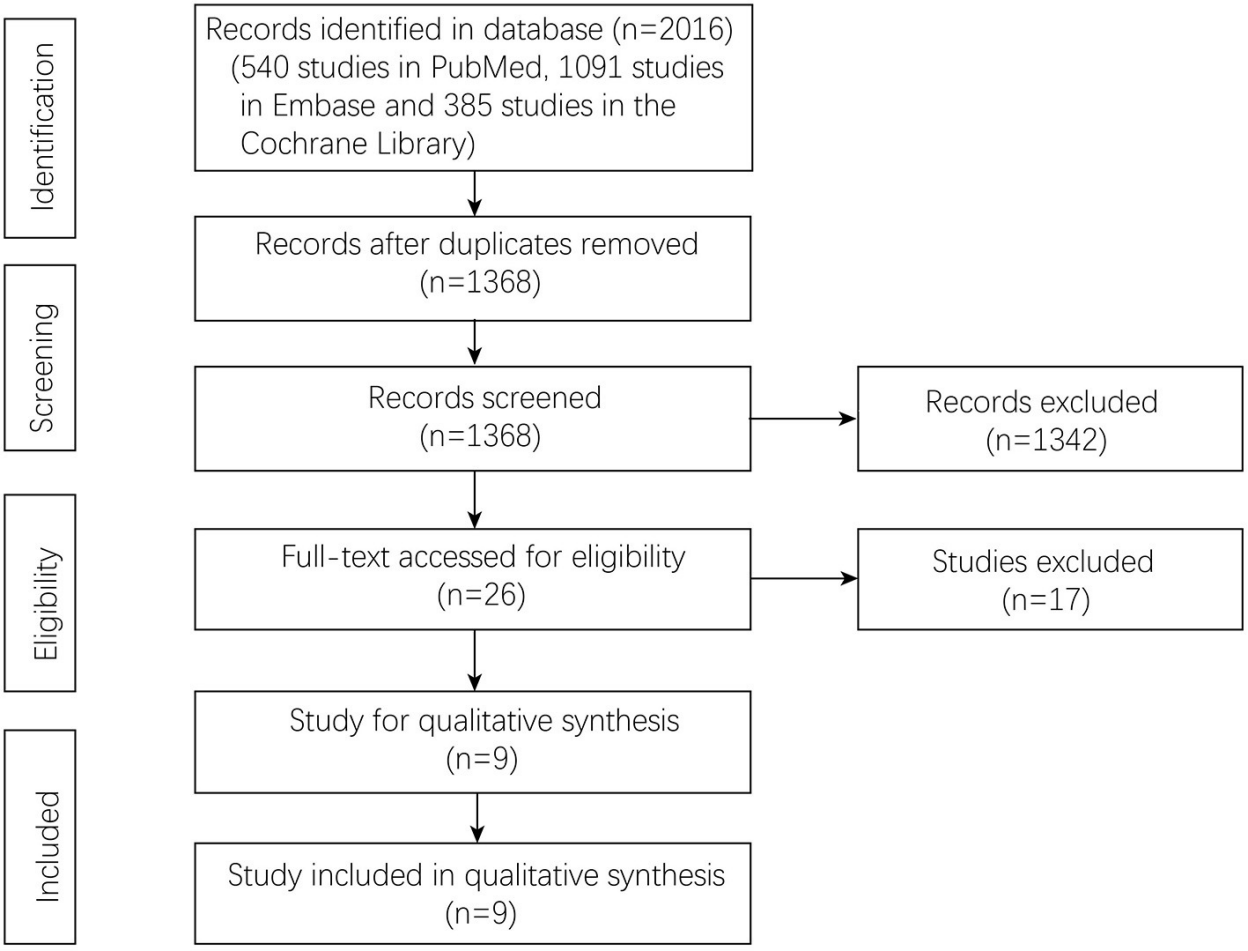
Flowchart of study selection.

A total of 2,016 studies were identified in the three databases, 540 studies in Pubmed, 1,091 studies in Embase, and 385 studies in the Cochrane Library. After removing duplicate studies, 1,368 studies were left for initial screening. After that, 26 studies were accessed with full text for potential eligibility. In total, nine studies were included in this meta-analysis, and the flow chart is shown in Figure 1.

### Patient Characteristics

Nine studies (13–16, 22–26) with a total of 6,541 patients were included in this meta-analysis. Five studies were from Japan, and four studies were from China. The year of publication was from 2001 to 2021, and all the studies were retrospective. The definition of CKD and non-CKD was according to eGFR, dialysis, and creatinine. Newcastle-Ottawa Scale (NOS) scores were accessed in each study and are shown in Table 1.

**Table 1:** Characteristics of the studies included in the meta-analysis.

**Author**	**Year published**	**Country**	**Study design**	**Study date**	**Sample size**			**Definition of CKD and Non-CKD**		**NOS**
					**CKD group**	**Non-CKD group**	**Total**	**CKD group**	**Non-CKD group**	
Yoshikawa T	2021	Japan	Retrospective	2011–2019	28	176	204	eGFR <45	eGFR ≥ 45	7
Ho SY	2021	China	Retrospective	2002−2017	43	129	172	Dialysis	Non-dialysis	8
Shirata C	2018	Japan	Retrospective	2002–2014	62	673	735	eGFR <45	eGFR ≥ 45	7
Yeh CC	2013	China	Retrospective	1996–2008	149	596	745	eGFR <15	eGFR ≥ 15	8
Hsu CY	2012	China	Retrospective	NA	502	1701	2203	eGFR <60	eGFR ≥ 60	8
Orii T	2008	Japan	Retrospective	1990–2006	17	51	68	CRF	Non-CRF	6
Toshima T	2012	Japan	Retrospective	1986–2009	17	705	722	Cr > 2	Cr ≤ 2	7
Yeh CN	2005	China	Retrospective	1982–2001	26	1198	1224	Dialysis	Non-dialysis	8
Cheng SB	2001	China	Retrospective	1989–1999	12	456	468	Dialysis	Non-dialysis	7

*CKD, chronic kidney disease; eGFR, estimated glomerular filtration rates, mL/min/1.73 m^2^; CRF, chronic renal failure; Cr, creatinine, mg/dl; NOS, Newcastle-Ottawa Scale; NA, not applicable*.

### Baseline Information

The baseline information included sex, age, tumor size, vascular invasion, intrahepatic metastases, liver cirrhosis and Child-Pugh classification, and was compared between the CKD group and the non-CKD group. After pooling all the data, the CKD group had a higher rate of Child-Pugh grade B than the non-CKD group (OR = 1.58, 95% CI = 1.3 to 1.93, *P* < 0.00001) (Table 2).

**Table 2:** Summary of characteristics between CKD group and Non–CKD group.

**Characteristics**	**Studies**	**Participants (CKD/ non–CKD)**	**Mean difference/odds ratio (95% CI)**	**Heterogeneity**
Baseline information
Male	9	856/5685	0.85 [0.72, 1.00]; *P =* 0.06	I^2^ = 0%; *P =* 0.75
Age, year	5	605/3784	0.80 [−6.69, 8.29]; *P =* 0.83	I^2^ = 99%; *P <* 0.00001
Tumor size, cm	4	103/2083	−0.36 [−1.76, 1.05]; *P =* 0.62	I^2^ = 89%; *P <* 0.00001
Vascular invasion	8	705/5041	0.86 [0.72, 1.02]; *P =* 0.09	I^2^ = 0%; *P =* 0.95
Intrahepatic metastases	3	96/1429	0.73 [0.40, 1.32]; *P =* 0.29	I^2^ = 0%; *P =* 0.85
Child–Pugh grade A	8	707/5089	0.53 [0.44, 0.63]; *P <* 0.00001	I^2^ = 0%; *P =* 0.86
Child–Pugh grade B	7	679/4913	1.58 [1.30, 1.93]; *P <* 0.00001	I^2^ = 8%; *P =* 0.37
Liver cirrhosis	5	266/2963	0.84 [0.64, 1.10]; *P =* 0.21	I^2^ = 0%; *P =* 0.54
Surgery–related information
Blood loss, mL	3	90/1954	−404.79 [−509.70, −299.88]; *P <* 0.00001	I^2^ = 0%; *P =* 0.67
Blood transfusion	4	256/2150	2.47 [1.85, 3.30]; *P <* 0.00001	I^2^ = 3%; *P =* 0.38
Hospital stay, days	3	60/1954	4.07 [−5.52, 13.67]; *P =* 0.41	I^2^ = 90%; *P <* 0.00001
Postoperative complications
Surgical site infection	5	223/1984	2.11 [1.02, 4.38]; *P =* 0.04	I^2^ = 16%; *P =* 0.31
Bile leakage	5	136/2061	1.60 [0.79, 3.27]; *P =* 0.19	I^2^ = 0%; *P =* 0.61
Liver failure	3	194/1477	1.50 [0.61, 3.70]; *P =* 0.38	I^2^ = 0%; *P =* 0.40
Pleural effusion	4	119/2010	2.74 [1.73, 4.32]; *P <* 0.0001	I^2^ = 22%; *P =* 0.28
Ascites	4	119/2010	1.94 [1.20, 3.13]; *P =* 0.007	I^2^ = 41%; *P =* 0.17
Postoperative bleeding	3	46/1212	4.55 [1.15, 17.96]; *P =* 0.03	I^2^ = 0%; *P =* 0.44
Short–term death	5	277/3099	2.28 [1.15, 4.53]; *P =* 0.02	I^2^ = 13%; *P =* 0.33

*CKD, chronic kidney disease*.

### Surgery-Related Information

The surgery-related information included blood loss, blood transfusion, and hospital stay. After pooling all the data, the CKD group had larger blood loss (MD = −404.79, 95% CI = −509.7 to −299.88, *P* < 0.00001) and a higher rate of blood transfusion (OR = 2.47, 95% CI = 1.85 to 3.3, *P* < 0.00001). However, no significant difference was found between the CKD group and the non-CKD group (MD = 4.07, 95% CI = −5.52 to 13.67, *P* = 0.41).

### Postoperative Complications

**Figure 2:**
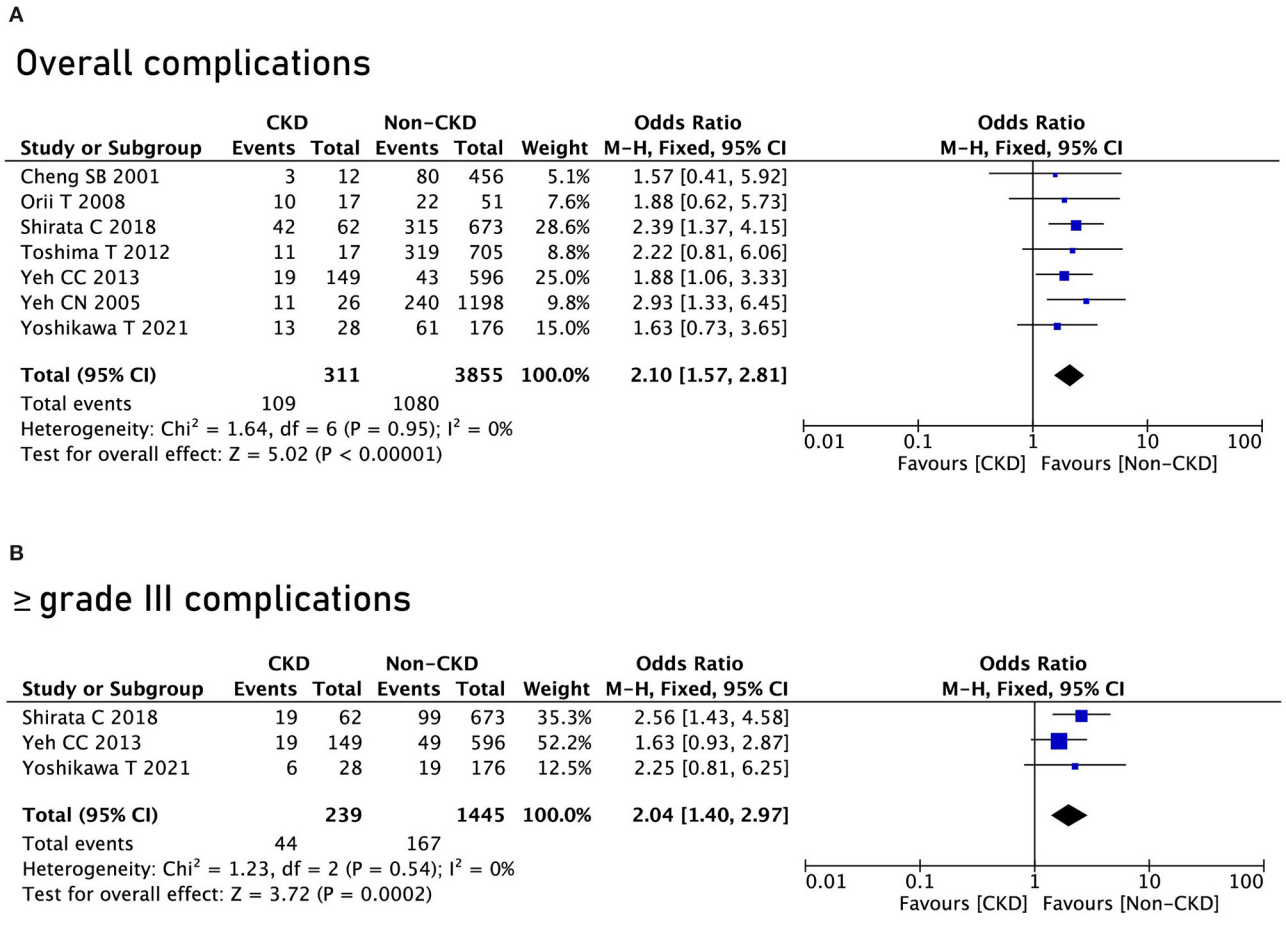
Complications between the chronic kidney disease (CKD) group and the non-CKD group. **(A)** Overall complications; **(B)** ≥ grade III complications.

Seven studies reported postoperative complications, and after pooling the data, the CKD group had a higher rate of overall complications (OR = 2.1, 95% CI = 1.57 to 2.81, *P* < 0.00001) (Figure 2A). In terms of ≥ grade III complications, the CKD group had a higher rate of ≥ grade III complications as well (OR = 2.04, 95% CI = 1.57 to 2.81, *P* = 0.0002) (Figure 2B).

As for specific complications, the CKD group had a higher rate of surgical site infection (OR = 2.04, 95% CI = 1.57 to 2.81, *P* = 0.0002), pleural effusion (OR = 2.04, 95% CI = 1.57 to 2.81, *P* = 0.0002), ascites (OR = 2.04, 95% CI = 1.57 to 2.81, *P* = 0.0002), postoperative bleeding (OR = 2.04, 95% CI = 1.57 to 2.81, *P* = 0.0002), and short-term death (OR = 2.04, 95% CI = 1.57 to 2.81, *P* = 0.0002) (Table 2).

### Overall Survival

**Figure 3:**
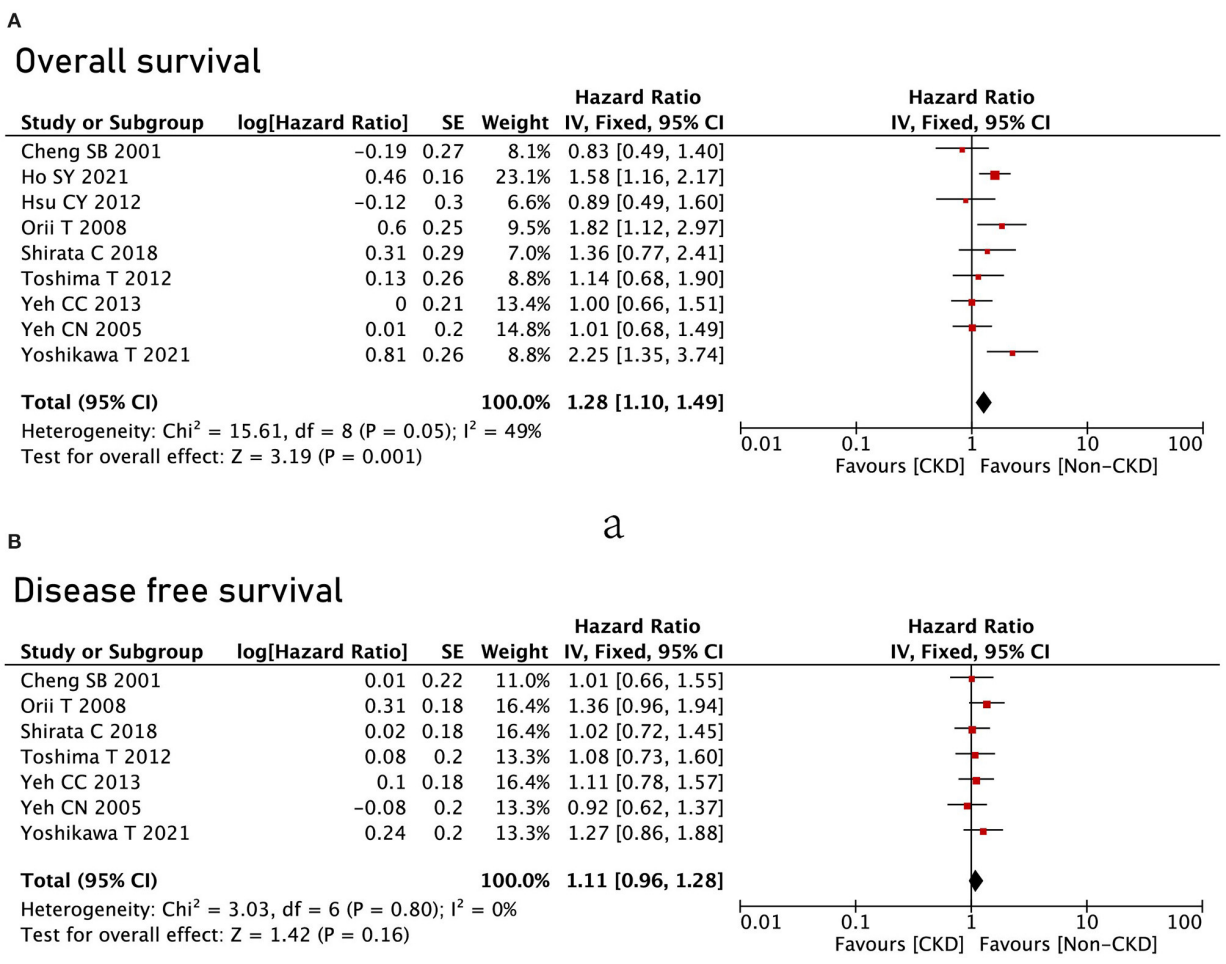
Survival analysis between the CKD group and the non-CKD group. **(A)** Overall survival; **(B)** disease-free survival.

All the nine studies reported OS between the CKD group and the non-CKD group, and the CKD group had poor OS compared with the non-CKD group (HR = 1.28, 95% CI = 1.1 to 1.49, *P* = 0.001) (Figure 3A). However, in terms of DFS, no significant difference was found between the CKD group and the non-CKD group (HR = 1.11, 95% CI = 0.96 to 1.28, *P* = 0.16) (Figure 3B).

### Sensitivity Analysis and Publication Bias

**Figure 4:**
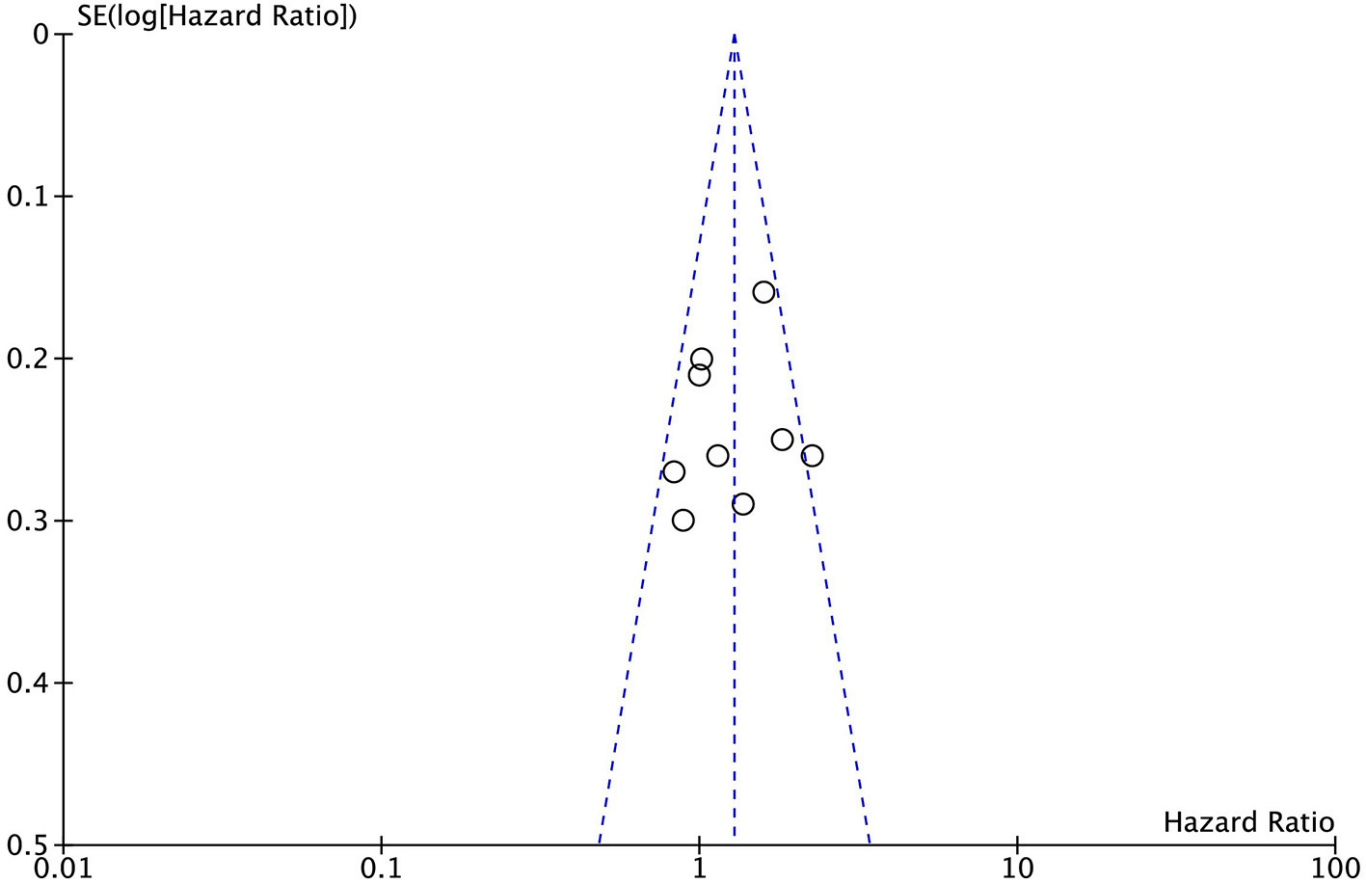
Funnel plot of overall survival.

Sensitive analysis was conducted by excluding one study in turns, and the results remained the same after excluding one study in turns. Publication bias was determined according to a funnel plot; the funnel plot was symmetrical visually (Figure 4).

## Discussion

A total of nine studies with 6,541 patients were included in this meta-analysis. After pooling all the baseline information, the CKD group had a higher rate of Child-Pugh grade B than the non-CKD group. As for the surgery-related information, the CKD group had larger blood loss and a higher rate of blood transfusion. In terms of complications, the CKD group had a higher rate of overall complications and a higher rate of ≥ grade III complications. The CKD group had poor OS compared with the non-CKD group; however, in terms of DFS, no significant difference was found.

As previously described, CKD has a certain impact on a variety of operations, including gastric cancer surgery (27), colorectal cancer surgery (28) and bladder cancer surgery (29). CKD could result in increased complications and reduced OS. However, in terms of liver resection for HCC, it remains controversial whether preexisting CKD has an impact on the complications and prognosis (13–16). Therefore, it is necessary for surgeons to explore whether there is a precise effect of CKD on liver resection for HCC.

In this meta-analysis, we found that postoperative complications were higher in the CKD group than in the non-CKD group. As for specific complications, we found that patients with CKD required more blood transfusions, and that the proportion of postoperative ascites was increased. The possible reason is that patients with CKD often suffer from other serious comorbidities, including diabetes and cardiovascular disease, which might inhibit wound repair and result in complications (15). In addition, because of nutritional deficiencies, loss of serum immune system components, and lymphocyte suppression, patients with CKD are in an immunosuppressive state; therefore, they might be more likely to suffer from postoperative infections and even death (30, 31).

There are many factors that could affect the prognosis after liver resection for HCC including presence of vascular penetration, satellite nodules, tumor differentiation, hepatitis, cirrhosis, and tumor staging (32, 33). In this meta-analysis, the CKD group had poor OS compared with the non-CKD group; however, in terms of DFS, no significant difference was found. The main reason was related to cardiovascular diseases. Patients with CKD had a higher risk of vascular disease and risk of events such as heart failure, myocardial infarction, stroke, atrial fibrillation, and peripheral arterial, after surgery (14, 15). Theses cardiovascular diseases might decrease OS. However, as for DFS, the main cause was the tumor itself; therefore, no significant difference was found.

Therefore, when patients with concurrent CKD and HCC are admitted to a hospital, examination of the progression of CKD is necessary. In addition, biological parameters including the immune system components and lymphocyte examination must be tested. For surgeons, it is important to reduce surgical time and give more attention to perioperative management.

There were some important data that were insufficient for meta-analysis and were presented in the included studies. Shirata et al. (24) reported that the CKD group had no effect on patients with Child-Pugh A liver function compared to the non-CKD group, and that poor OS was identified in patients with concurrent CKD and Child-Pugh B liver function. Hsu et al. (13) reported that CKD could result in a poor outcome of patients who underwent TACE. Another study did propensity score matching (PSM) and found that the CKD group had poor prognosis, (23) the baseline information had no significant difference after the PSM, and more studies with the PSM method were needed in the future.

There were some limitations that existed in this meta-analysis. First, only nine retrospective studies were included in this study, which was relatively small. Second, countries of the included studies were restricted to China and Japan; therefore, the results could only be applied to eastern Asians. Third, the definition of CKD group and non-CKD group was different, which might result in heterogeneity. Fourth, the included studies ranged from 20 years ago to now. Improvements in primary postoperative care of the patients during these two decades might have reduced mortality and morbidity; thus, bias might occur. Therefore, multicenter, high-quality randomized controlled trials should be performed in the future.

In conclusion, preexisting CKD was associated with higher ratio of complications and poor OS. However, no difference was found in terms of DFS.

## Data Availability Statement

The raw data supporting the conclusions of this article will be made available by the authors, without undue reservation.

## Ethics Statement

Ethical review and approval was not required for the study on human participants in accordance with the local legislation and institutional requirements. The patients/participants provided their written informed consent to participate in this study.

## Author Contributions

X-YL and Z-QZ: data extraction. C-YW: quality assessments. C-YW and X-YL: data analysis and writing (original draft). C-YW, X-YL, Z-QZ, Y-XC, WT, BZ, and CY: writing (review and editing). All authors read and approved the final version of the manuscript.

## Conflict of Interest

The authors declare that the research was conducted in the absence of any commercial or financial relationships that could be construed as a potential conflict of interest.

## Publisher's Note

All claims expressed in this article are solely those of the authors and do not necessarily represent those of their affiliated organizations, or those of the publisher, the editors and the reviewers. Any product that may be evaluated in this article, or claim that may be made by its manufacturer, is not guaranteed or endorsed by the publisher.
